# Emotional Intelligence among Female Nursing Leaders in a Transformational Era

**DOI:** 10.1155/2024/9973339

**Published:** 2024-04-20

**Authors:** Noura Almadani, Majed Alamri

**Affiliations:** ^1^Community and Psychiatric Mental Health Nursing Department, College of Nursing, Princess Nourah bint Abdulrahman University, Riyadh 84428, Saudi Arabia; ^2^Nursing Department, College of Applied Medical Sciences, University of Hafr Al Batin, Hafar Al Batin 39524, Saudi Arabia

## Abstract

**Background:**

Emotional intelligence (EI) is an instrumental quality for effective management in the changing landscape of healthcare leadership, specifically among female nursing leaders.

**Aim:**

This study aims to assess the EI among female nursing leaders in Saudi Arabian hospitals and to examine its connection with leadership and effectiveness of decision-making during a transformational period.

**Methods:**

This study applied a correlational descriptive cross-sectional methodology to gather data from 232 female nursing leaders. The data were collected via an online survey using convenience sampling. The study incorporated demographic data as well as a 16-item EI scale. Approval was granted by the ethics committee, and the participants' privacy was appropriately ensured.

**Results:**

The majority of the participants were experienced professionals aged 25 years and above, with a significant proportion holding a bachelor's degree or higher, and over five years of leadership experience. The study revealed a positive connection between EI and self-leadership (self-awareness, self-reflection, and self-motivation), especially among leaders with more experience. An investigation considering many variables revealed a noteworthy model that explains 55.2% of the variation in EI scores. This model portrays that higher self-leadership scores, as well as longer experience, are predictors of higher levels of EI.

**Conclusions:**

This study has found that EI is widespread among female nursing leaders, and it is meaningfully and positively associated with their aptitude for self-leadership. The fact that more leadership experience correlated with higher EI signifies the need for focused EI development programs in the nursing leadership curriculum. These insights are instrumental for developing leadership that can effectually manage the intricacies of the evolving healthcare transition.

## 1. Introduction

All nurse leaders must possess a comprehensive range of skills to effectively lead in this transformational era [1]. The transformational era of healthcare refers to a period of significant changes and advances in the industry, such as technological advances, new treatment modalities, and shifting patient demographics [2]. Emotional intelligence (EI) is particularly important for female nursing leaders in this era as it equips them with the skills to navigate these changes, inspire their teams, and foster a culture of continuous learning and growth. By incorporating EI into their mindset, female nursing leaders can effectively lead their teams through the challenges and complexities of healthcare transformation, ultimately ensuring the delivery of high-quality patient care and to maintain a positive work environment [2]. To be effective, it is essential for female nursing leaders to be aware of their emotions and manage them. Female nursing leaders often face unique challenges in the workplace, such as gender-based discrimination and pay inequality [3].

As a result, it is important for female nursing leaders to be aware of their own emotions to manage their stress and stay emotionally healthy [1]. By understanding their emotions, female nursing leaders can develop strong coping strategies and be better equipped to navigate the challenges they may face in the workplace. This awareness of their emotions can also help to better empathize with and support their colleagues, regardless of gender. Moreover, the literature supports that EI has significant influence on authentic leadership [4]. EI stems from the concept of social intelligence, which refers to the ability to understand and influence other people [5].

For instance, a female leader with high EI would recognize when a team member is feeling overwhelmed and provide support to that team member rather than reprimanding them for not achieving a goal. In addition, several studies have demonstrated that EI can also be used to foster team spirit, create a culture of mutual respect and trust, manage staff effectively, and make decisions based on empathy and understanding [4]. Female leaders tend to have a stronger awareness of their emotions and are better able to recognize and empathize with their colleagues' emotions. This often leads to better communication and problem-solving skills, which can be beneficial when working with male colleagues. In addition, females may be more likely to offer emotional support and be more understanding of their colleagues' needs [5]. EI analogous to a compass that guides decisions and actions, helping leaders to stay on course and guide the team in achieving its goals.

Gender and leadership have, in recent times, been at the center of controversy [6]. As more females occupy leadership roles in many countries, questions arise as to whether they should lead in specific areas and whether both males and females exhibit similar leadership traits [7].

The literature shows that gender differences do exist and affect leadership roles in certain circumstances [5, 6]. For example, a report by the Pew Research Center indicates that females only account for 24% of the total members of national legislative bodies globally [8].

Furthermore, a meta-analysis by Lopez-Zafra et al. on transformational leadership, EI, and gender stereotypes revealed that females exhibited higher charisma, transformational characters, and EI skills than males, despite traditional stereotypes of females in leadership [5]. For example, women are often stereotyped as not aggressive enough for leadership positions. They are viewed as caregivers, where men are viewed as assertive. According to a recent report, the unemployment rate for Saudi females has declined to 15.4% in the fourth quarter of 2022 from 2021, 2020, and 2019, with the leading indicators of the Women's Labor Force Survey showing a marked improvement [9]. The percentage of employed females increased to 30.4% from 27.6% in the fourth quarter of 2021, due to the decline in female unemployment, the expansion of their economic participation, and the growth of their employment in a variety of fields. It increased slightly from 35.6% in the fourth quarter of 2021 to 36% in the fourth quarter of 2022 [9]. Leadership styles are essential variables that may explain gender differences in leadership. Different leadership styles require different skillsets, and females may be more likely to have the necessary skills to lead effectively [4, 5]. For example, females may be more likely to have empathy and interpersonal skills, which are important in leading a team. In addition, females may be more likely to make decisions based on the best interests of the team rather than on personal gain. Yet, different leadership styles may require different skills, such as the ability to motivate and inspire others [5], which are typically associated with males. Females who are successful in leadership roles may have developed these skills through different experiences and methods than males.

An effective leadership style includes demonstrating higher EI. EI influences an individual's establishment as a role model, which helps to gain the trust of team members [4]. EI is an inherent attribute, regardless of gender or sex. However, the level of EI varies among individuals and can often be the difference between influential leaders and less successful ones. Multiple studies show that females score higher than males in EI and are generally more expressive than males [3, 5]. This means that females tend to be more supportive and can effectively manage emotions. Characteristics such as femininity and emotional clarity are essential predictors for transformational leadership [5]. Emotional clarity, specifically, is an important predictor of intellectual stimulation and inspirational charisma in the workplace. Emotional clarity is the ability to identify, understand, and communicate emotions accurately and effectively. It involves self-awareness, self-regulation, and self-expression and is an important skill for leaders to be successful [6, 7].

According to the literature, EI has become a key characteristic of female nursing leaders in the current healthcare transformation landscape [8, 9]. The purpose of this study is to assess the EI of female nursing leaders in the healthcare sector. The results of the study provide insight into how EI can be improved among female nursing leaders to foster better decision-making and enhance leadership skills. As a result of strengthening these skills, patient care and outcomes are likely to improve.

## 2. Methods

### 2.1. Study Design and Participants

A correlational descriptive cross-sectional design was conducted using online survey questionnaires.

### 2.2. Sampling and Data Collection

A total of 232 female nursing leaders working in government and nongovernment hospitals in Saudi Arabia were selected using convenience sampling. Using power analysis to determine the sample size to achieve a significance level of 0.05 and power of 0.80, the minimum requirement was 143 subjects. Data were collected through the online survey questionnaires and the responses were confidential. The survey questionnaires were designed to be short and easy to complete to encourage participation.

### 2.3. Eligibility and Exclusion Criteria

A nurse with a leadership experience of five years or more (head/charge or equivalent, administrator or equivalent, and supervisor or equivalent) was eligible to participate in the study. Nurses without leadership experience and male nurses were excluded.

### 2.4. Instruments/Measurements

To gather information to assess the EI among female nursing leaders in the healthcare sector during the transformation era, the Wong and Law Emotional Intelligence Scale (WLEIS) five-point Likert scale with categories of 1–5 (1 = strongly disagree; 5 = strongly agree) was used in this study [10]. The WLEIS contains 16 items based on the ability model. It has four dimensions: self-emotional appraisal, other emotional appraisal, using emotions, and regulating emotions. The total EI score is equal to the mean of the items 1–16. The total self-emotions appraisal is based on the mean of items 1–4. The total regulation of emotions is equal to the mean of items 5–8. Total use of emotion is equal to mean of items 9–12. Total others emotion appraisal is equal to the mean of items 13–16. Increased levels of EI correspond to higher scores on the scale. The scale's factor loadings represent different subconstructs of EI, and each factor has items that measure specific facets of EI. The correct interpretation of scores calls for the reflection of the highest and lowest possible scores on the scale and the knowledge of the meanings of each factor loading [10]. This measure has been cited over 1300 times by Google Scholar citation searches, demonstrating its popularity among researchers [11]. Based on an earlier study conducted in Saudi Arabia, the final scale had an overall Cronbach's alpha of 0.81, adequate construct validity, and a high content validity index, which indicates excellent internal consistency [12]. There was also a section on demographic characteristics including age, nationality, years of experience, level of education, work experience, workplace, sector, current position, and training courses in the past year.

### 2.5. Statistical Analysis

The statistical data were coded and entered into the statistical package of social science (SPSS), version 23. Descriptive statistics were generated for all study variables. Pearson correlations, polyserial correlations, and regression analysis were computed to examine the intercorrelations among study variables.

### 2.6. Ethical Considerations

Ethical approval for this study was obtained from the Institutional Research Ethics Committees (IRB number: HAP-01-R-059). Written consent from study subjects was obtained. Confidentiality of the data and privacy of the study subjects was maintained. Subjects were assured they had the right to withdraw from the study.

## 3. Results

### 3.1. Participants' Characteristics

A total of 232 female nursing leaders completed the survey; their demographic characteristics are summarized in Table 1. The majority of participants were aged 25 years and above (88.8%). Majority were Saudi nationals (64.2%) and had more than 5 years of leadership experience (61.6%). A majority of the participants held a bachelor's degree or higher (85.4%). Participants had a variety of workplace expertise. A majority of the participants did not provide a specific designation, choosing the option of “others” (52.2%). A majority of the participants had taken self-development courses in the previous year (73.7%) and a majority worked in the government sector (80.6%).

### 3.2. EI and Its Factor Scores in Female Nursing Leaders

The mean values of EI total score and factor scores of self-emotions appraisal, regulation of emotions, use of emotion, and other emotions appraisal were 4.00 ± 0.56; 4.03 ± 0.69; 4.03 ± 0.71; 4.12 ± 0.65; and 3.84 ± 0.70, respectively (Table 2). These results suggest that participants generally had moderate to high levels of EI, as indicated by the mean scores falling within the four-point range. The highest mean score was observed for the factor of regulation of emotions, indicating that participants were particularly skilled at managing and controlling their emotions. In addition, the high standard deviations indicate variability in the participants' responses, highlighting individual differences in EI within the study cohort.

### 3.3. Self-Leadership Scale and Its Factor Scores in Female Nursing Leaders

The mean values of self-leadership scale total score and factor scores of behavior awareness and volition, task motivation, and constructive cognition were 36.67 ± 4.93; 12.65 ± 1.87; 11.99 ± 2.05; and 12.03 ± 1.94; respectively (Table 3). These findings suggest that, on average, participants had moderate levels of self-leadership, with higher scores in behavior awareness and volition. Task motivation and constructive cognition scores were also relatively high, indicating a strong sense of motivation and cognitive strategies for self-leadership among the participants.

### 3.4. Multivariate Analysis: Multiple Linear Regression-Associated Factors of EI

The multiple regression model was statistically significant, *F* (17, 217) = 17.451, *p* < 0.001, *R*2 = 0.552 (Table 4). For every unit change in the self-leadership score, the EI level increased by 0.72 (*p* < 0.001). The highest level of experience category (>25 years) showed a higher level of EI 0.205 (*p*=0.021). These findings indicate a significant positive relationship between self-leadership and EI, with a 0.72 increase in EI for every unit change in the self-leadership score. Furthermore, the highest level of experience category (>25 years) was associated with a statistically significant higher level of EI, suggesting that years of experience may contribute to higher levels of EI. These results highlight the importance of self-leadership and experience in fostering EI.

## 4. Discussion

The findings of this study demonstrate the interconnected nature of EI among nursing leaders during a transformation period. It is indicated that female nursing leaders who have high EI scores have great potential to be effective leaders since they are likely to practice EI in their leadership style. Subsequently, EI leadership guides the formation of highly cohesive groups. There is an abundance of empirical findings supporting the relationship between EI and effective leadership styles [1, 4, 5, 10, 13]. The study findings clarified this association. Specifically, there are two key components of EI: emotional clarity and emotional regulation. For example, the findings indicate that female nursing leaders who are aware of their team members' emotions are better leaders because they promote optimal participation by the team members.

According to research by Mysirlaki and Paraskeva, females demonstrate EI in their leadership more than males [14]. Furthermore, individuals with higher EI are more likely to adopt an effective leadership style that is required in the current transformational era [14, 15]. Yet, a study has shown that females tend to possess higher levels of EI than males, making them more attuned to the emotions and needs of others [16]. This heightened EI can greatly benefit female nursing leaders in healthcare, allowing them to effectively connect with and support their teams, as well as navigate the complex and evolving challenges of the organization. These findings help explain the evidenced higher levels of EI among female nursing leaders who were subjects in this study. The correlation between higher levels of EI and nursing leadership is underlined by the four elements of leadership styles, which include idealized influence, inspirational motivation, intellectual stimulation, and individual consideration. These elements collectively contribute to the effectiveness of leaders who possess high EI [17].

Nursing leaders are required to demonstrate to their staff that they are hands-on in the directives they propose [18]. This attribute encourages commitment among the team and allows nursing leaders to put their advocacy into practice. In this transformative era, female leaders need to demonstrate idealized influence whereby they act as exemplary role models. This approach to leadership means acting as a role model—a strategy that further contributes to higher levels of trust and confidence from the rest of the team [17–20]. An emotional relationship created between the leader and the team because the leader applies emotional support and spreading their individual emotions concerning how they expect their followers to behave. A mutual scenario of emotional manifestation is also created, such that the followers also demonstrate emotional commitment that fosters collaboration in a team [18, 19].

EI also helps nursing leaders demonstrate certain nonverbal emotional cues that create the perception of a charismatic and effective leader. For instance, simple cues such as eye contact go a long way in establishing healthy working relations between a leader and the team [1, 4]. One of the scores' findings that demonstrated effective teamwork and collaboration in the study participants was “others emotion appraisal.” As the participants achieved high scores in this category, one can infer better levels of interpersonal functioning because these nursing leaders are likely to display appropriate reactions to the various personalities on the team. By understanding and tailoring reactions to the different emotional presentations that various individuals demonstrate, a nursing leader generates the emotional knowledge of how to gain optimum productivity and cooperation from staff members [10].

Effective female nursing leadership includes streamlining the staff toward attaining a common goal. This involves monitoring not only the usual protocol measures but also challenging scenarios, such as change implementation [10–12]. During change implementation, a significant number of staff may demonstrate resistance to change, which can lead to frustration and negative emotions on the part of the leader. Even so, nursing leaders realize that attaining their envisioned goal requires optimum input from every stakeholder. Such challenging scenarios are significant determinants of a nursing leader's ability to maintain optimum functionality amid stressful conditions, which is why emotional regulation is a significant attribute of a successful leader [12–15].

EI can assist nursing leaders navigate the challenges of implementing change and working under pressure. Leaders with EI create a work environment marked by enthusiasm and flexibility, which, ultimately, supports their key goal of implementing innovation and optimizing productivity [13]. Such leaders are better equipped to create this work environment because they have immersed themselves in the activities they are proposing to their team. This scenario was evident in the study as the female nursing leaders achieved high scores of self-leadership. An ability such as this implies that the leader proposes expectations on which she can also deliver and understands if some members of the team find the challenge difficult to achieve.

EI is an integral aspect of maintaining group cohesiveness so that members work toward a common goal despite individual differences [13–15]. Building group cohesiveness, therefore, leads to high levels of individual and team engagement. There is an abundance of the literature demonstrating improved performance and relationships among cohesive groups [16]. Members of a cohesive team also display higher satisfaction levels with their performance because they feel they have been given the opportunity to provide an optimum contribution to the course of leadership. In the transformative era, leaders achieve this goal when they not only act as role models but also foster intellectual stimulation to the group members [12].

Research on effective leadership styles has demonstrated improved group outcomes when more inclusive leadership styles are employed, as opposed to those that bestow all the power on the leader [12, 13]. It is crucial for a leader to encourage feedback from individual team members as they may have useful insights derived from their specific experiences in their roles [14]. In as much as a leader may hold a vision for a given course, maximum contribution from the rest of the team is necessary to determine the effectiveness with which the goal is realized. Accepting input from team members makes them feel that that their contribution is valued [11]. EI allows leaders to fuel individual team members' contribution through task motivation, as indicated by the high scores achieved in the study cohort. One method to attain task motivation is through valuing members' contributions and feedback because this allows members to generate a self-driven desire for intellectual stimulation [12]. As such, the female nursing leader's initial vision becomes the team members' challenge to explore every method of achieving the goal.

EI enables leaders to foster innovation and creativity among the team members because they are free to develop a critical approach toward the status quo and apply problem-solving techniques to challenges [13]. The high scores achieved by the participants' task motivation imply that the cohort possessed the skills necessary to motivate team members toward a given course. Leaders with high task motivation ability demonstrate skills such as active listening, encouraging team members to communicate and participate, adaptability, and being supportive.

Effective leadership skills are among the several clinical competence areas built by experience [13]. As such, transitioning from a novice to an experienced nurse comes with the benefit of progressively gaining competence in leadership. As nurses spend many years in a clinical area, they become better at establishing cohesion and collaboration in the workplace, with a leader's perspective [14].

The study findings showed higher levels of EI in female nursing leaders who had more years of workplace experience (>25 years). The clinical area can be challenging in terms of applying theoretical knowledge in practice. It often takes time to attain practice competence, even for a nurse who demonstrates excellence in theoretical knowledge. The advent of nursing theories such as Patricia Benner's theory of transition from novice to expert nursing provides sufficient proof of the journey a nurse undertakes to attain optimum competence levels in clinical roles [15]. Evidence from studies also demonstrates nurses' improved competence and confidence levels in their clinical skills, such as catheterization, injection, and sample collection [16].

Similarly, the area of nurse leadership is largely built by years of clinical experience. This concept has also introduced the vital role of nurse mentors in fostering professional growth for novice nurses. Nurse mentors aid novice nurses in avoiding specific errors that are common among new graduates [17]. They also provide insightful guidance on strategies of successful nurses in clinical areas such as workflow management, networking, and workplace collaboration [18]. Experience gives nurses hands-on skills with improved efficiency and the confidence to handle even challenging scenarios, such as ethical dilemmas [19]. This evidence implies that nurse leadership skills will also improve progressively with time and experience.

Through the study by Benner, significant findings were discovered that are related to the relationships between years of experience, EI levels, and the demonstration of leadership skills among nurse leaders. It is noteworthy that nurses with at least five years of experience have exhibited more comprehensive nurse leadership competence compared to novice nurses. This remark illustrates the importance of practical experience to develop leadership skills in the nursing field. Nurses with many years of experience are given an opportunity to hone essential skills such as decision-making, communication, and team management, which are key to good leadership. In addition, it was found that nursing leaders with over 25 years of work experience were those who had higher levels of EI. This reveals that prolonged professional tenure contributes to the maturing and perfecting of emotional regulation and awareness skills, which are crucial to successful leadership. The sense that more time is spent in different types of clinical environments and interpersonal relationships increases the EI that nurse leaders with lengthy experience possess. Overall, these findings reinforce the need to pay attention to and utilize the complementary nature of experience, EI development and leadership proficiency in the process of building a cadre of skillful nurse leaders able to lead healthcare organizations through their complexity [20]. For instance, effective communication is a crucial aspect of nurse leadership that helps establish better relationships and succinctly present ideas [21].

More experienced nursing leaders also possess better decision-making and critical thinking skills that enable them to analyze complex situations and evaluate options to determine the best course of action [22, 23]. Furthermore, having analytical and problem-solving abilities assists seasoned nurses respond to situations more effectively by adopting novel approaches and putting into practice tried-and-true tactics that are supported by data and experience. For example, evidence-based practice is a strategy designed to ensure nurses provide high-quality care [23].

In addition, for experienced nurses, the application of evidence-based practice includes the adoption of leadership styles, qualities, and skills that have evidence indicating quality outcomes. These nurses also have strongly established EI that has been activated by prolonged durations of self-awareness as well as the knowledge of how different people react [24]. There is also strong literature backing the positive impact EI has on effective conflict resolution, team inspiration, and maintaining a supportive work environment [25–27].

Therefore, apart from gaining knowledge on effective leadership strategies, practical experience assists nurses to become great leaders through team management and operational decision-making. In this transformative era, female nursing leaders must acquire skills beyond balancing costs, monitoring productivity, and maintaining high levels of patient and staff satisfaction [28]. They also need to act as role models and influence processes at not only the organizational but national levels as well. A strong nursing leader motivates team members, creates a safe and collaborative work environment, and ultimately boosts morale and job retention [12].

### 4.1. Limitations

A significant limitation of the study is that the study participants were only selected from Saudi Arabian hospitals. This sampling method represents subjects from a specific region. Considering leadership skills are influenced by factors such as culture, the sampling process may have been biased in this regard. Therefore, the findings cannot be generalized to participants from other regions with different cultural backgrounds. The sample size was also relatively small for the generalizability of the findings to larger settings. Another limitation of the study was that only female participants were included. This prevented a comparison of females to males, as leaders who are just as effective as or more effective than men. This finding would have assisted in demystifying the misconception of the inability of females to lead that has led to leadership stereotyping based on gender.

### 4.2. Recommendations

To display their mastery of leadership abilities in this transformative period, leaders must receive training in EI. Leaders also must embrace leadership styles that encourage team members. Building EI is an inner motivation that passionate nurses should cultivate since it greatly enhances cohesiveness and participative leadership. There is a need for leaders to not only understand and control their own emotions but also understand other people's emotions. To apply these findings to the clinical setting, hospitals must create EI models that can include specific personality assessments, behavioral evaluations, and other relevant factors to ensure that the chosen leaders possess the necessary emotional stability for the clinical setting. Hospitals may start putting the results into practice by launching leadership development programs designed specifically for nursing leaders; these programs should emphasize resilience in terms of managing one's own emotions as well as comprehending those of others. To reinforce EI in nursing leaders, hospitals can also develop slogans that frequently remind leaders of the importance of this quality. Examples of such slogans could include “Empathy is Essential,” “Lead with Compassion,” “Leading with Emotional Intelligence,” “Creating a Caring Culture,” or “Empowering through Understanding.” These slogans can be printed and mounted at strategic locations to serve as vital tools of communication tool to this course.

### 4.3. Implications to Nursing Management

The research on EI in the transformational era among female nursing leaders has several implications for nursing management. Firstly, it highlights the importance of nurturing EI skills in nurses, as it can positively impact their leadership abilities and the overall quality of patient care. Secondly, it emphasizes the need for healthcare organizations to prioritize the recruitment and development of emotionally intelligent leaders who can effectively navigate the complexities of the healthcare landscape. Lastly, the findings suggest that incorporating EI training into nursing leadership programs can be a valuable investment in enhancing the effectiveness and success of nursing management. By understanding and addressing these implications, nursing management can foster a more inclusive and empowering environment for female leaders, ultimately enhancing the overall quality of patient care.

## 5. Conclusion

By assessing EI levels in female nursing leaders, this study sought to establish EI as a forerunner to a transformational leadership style. As established from the study's findings, participants showed high levels of manifestation of transformational behavior. It has also been established from the literature that EI is critical in predicting transformational leadership based on the key components of emotional clarity and emotional regulation. The findings have also challenged the common misconception that females are not effective nurse leaders. As established, individual behavior is determined by factors that are not limited to gender but also to other determinants such as environment, personal experiences, culture, and heredity. The study's findings point to the necessity of creating instruments to raise the EI of nursing leaders to achieve transformational leadership. Furthermore, after receiving sufficient training in EI, leaders, male and female alike, can demonstrate EI in their leadership style to achieve greater levels of employee satisfaction and organizational performance.

## Figures and Tables

**Table 1:** Characteristics of female nursing leaders in transformational era.

Characteristics	Mean ± SD/number (percentage)
Age (in years)	
<25 years	26 (11.2%)
25–35 years	94 (40.5%)
36–45 years	73 (31.5%)
>45 years	39 (16.8%)
Nationality	
Saudi	149 (64.2%)
Expatriates	83 (35.8%)
Years of experience	
<5 years	89 (38.4%)
6–15 years	53 (22.8%)
16–25 years	25 (10.8%)
>25 years	65 (28.0%)
Educational level	
Diploma/ADN	34 (14.7%)
Bachelor's degree	126 (54.3%)
Master's degree	41 (17.7%)
Doctoral degree	31 (13.4%)
Workplace	
Coronary care unit	2 (0.9%)
Education	35 (15.1%)
Emergency	28 (12.1%)
Intensive care unit	38 (16.4%)
Medical	26 (11.2%)
Other	81 (34.9%)
Pediatric	6 (2.6%)
Surgical	16 (6.9%)
Current position	
Administrator (or equivalent)	44 (19.0%)
Head/charge (or equivalent)	47 (20.3%)
Others	121 (52.2%)
Supervisor (or equivalent)	20 (8.6%)
Self-development courses in last 12 months	
No	61 (26.3%)
Yes	171 (73.7%)
Work sector	
Government hospitals	187 (80.6%)
Other government sectors	14 (6.0%)
Private	31 (13.4%)

SD: standard deviation.

**Table 2:** Emotional intelligence and its factor scores in female nursing leaders.

	Minimum	Maximum	Mean	SD
Total self-emotions appraisal	1.00	5.00	4.03	0.690
Total regulation of emotions	1.00	5.00	4.033	0.709
Total use of emotion	1.00	5.00	4.123	0.649
Total other -emotion appraisal	1.25	5.00	3.843	0.699
Total emotional intelligence	1.13	5.00	4.003	0.559
Valid *N* (listwise)				

SD: standard deviation.

**Table 3:** Self-leadership questionnaire and its factor scores in female nursing leaders.

	Minimum	Maximum	Mean	SD
Behavior awareness and volition	4.00	15.00	12.65	1.87
Task motivation	3.00	15.00	11.99	2.05
Constructive cognition	3.00	15.00	12.03	1.94
Total score	14.00	45.00	36.67	4.93

SD: standard deviation.

**Table 4:** Multiple regression predictors of the emotional level in female nursing leaders.

Independent variable	*β*	SE	*T* values	*p* values	Model unadjusted *R*2; adjusted *R*2; *p* value
Self-leadership score	0.720	0.005	14.939	<0.001	0.586, 0.552, <0.001
Age categories					
Age: <25 years	Ref				
Age: 25–35 years	0.145	0.102	1.599	0.111	
Age: 36–45 years	0.057	0.128	0.529	0.597	
Age: >45 years	−0.108	0.165	−0.956	0.340	
Highest education level					
Diploma/ADN					
Bachelor's degree	−0.034	0.078	−0.482	0.630	
Master's degree	0.009	0.090	0.140	0.889	
Doctoral degree	−0.077	0.112	−1.138	0.257	
Nationality					
Saudi	Ref				
Expatriates	0.091	0.067	1.562	0.120	
Experience in years					
Less than 5 years	Ref				
6–15 years	0.011	0.084	0.153	0.879	
16–25 years	0.076	0.117	0.848	0.397	
>25 years	0.205	0.155	2.332	0.021	
Current position					
Other	Ref				
Head/charge (or equivalent)	0.027	0.069	0.536	0.592	
Administrator (or equivalent)	−0.071	0.080	−1.266	0.207	
Supervisor (or equivalent)	0.071	0.096	1.443	0.151	
Self-development courses in the last 12 months					
No	Ref				
Yes	0.043	0.062	0.882	0.379	
Work sector					
Private	Ref				
Other government organizations	−0.006	0.127	−0.111	0.912	
Government hospitals	0.015	0.075	0.281	0.779	
Intercept	0.851^*∗*^	0.227	3.741	<0.001	

^*∗*^Unstandardized coefficient for intercept, for all other independent variables standardized beta coefficient are shown. SE: standard error.

## Data Availability

The data used to support the findings of this study are available from the corresponding authors on request.
